# Room for Improvement: A 20-Year Single Center Experience with Allogeneic Stem Cell Transplantation for Myelodysplastic Syndromes

**DOI:** 10.1007/s12288-021-01508-8

**Published:** 2021-12-10

**Authors:** Katarzyna Duda, Agata Wieczorkiewicz-Kabut, Adrianna Spałek, Anna Koclęga, Anna J. Kopińska, Krzysztof Woźniczka, Grzegorz Helbig

**Affiliations:** Department of Hematology and Bone Marrow Transplantation, Medical School of Silesia, Silesian Medical University, Dąbrowski Street 25, 40-032 Katowice, Poland

**Keywords:** Allogeneic stem cell transplantation, Graft versus host disease, Ferritin, Myelodysplastic syndromes, Overall survival

## Abstract

Allogeneic stem cell transplantation (allo-SCT) remains the only curative therapeutic approach for patients with myelodysplastic syndromes (MDS). The aim of the study was to assess the efficacy/safety of allo-SCT as well as to identify factors influencing post-transplant survival. One hundred and two MDS patients (median age: 48 years; 57 males) who underwent allo-SCT were retrospectively evaluated. Twenty seven patients were transplanted from HLA-matched sibling and 75 patients received grafts from unrelated donors. Peripheral blood was a source of stem cell for 79 patients. Reduced intensity conditioning was used in 64 subjects. Acute and chronic graft versus host disease (GvHD) developed in 61 and 19 of patients, respectively. In total, 61 patients have died. The causes of deaths included infectious complications (n = 30), steroid-resistant GvHD (n = 17), MDS relapse (n = 9) and transformation to AML (n = 5). Non-relapse mortality and cumulative incidence of relapse at 2 years were 49.8% and 9%, respectively. 41 patients are alive at last contact and present full donor chimerism. 38 patients remain in complete hematological remission (CHR), 3 patients had CHR with incomplete platelet recovery. Median follow-up from diagnosis of MDS and transplantation are 27.1 months and 7 months respectively. Overall survival and relapse-free survival were 41% at 2 years. Increased serum ferritin level > 1000 ng/ml, presence of acute GvHD, grades III–IV acute GvHD and high hematopoietic cell transplantation-comorbidity index were found to negatively influenced survival. Allo-SCT for MDS is feasible procedure with a proportion of patients to be cured.

## Introduction

Myelodysplastic syndromes (MDS) are a heterogeneous group of clonal hematopoietic disorders characterized by peripheral blood cytopenias and increased risk of progression to acute myeloid leukemia (AML). The course of MDS is highly variable—from indolent subtypes with long-term survival to the cases with very poor prognosis and rapid transformation to AML [1]. The prognosis of patient with MDS can be evaluated using various scoring systems—the most common are the International Prognostic Scoring System (IPSS) and its revised variant—IPSS-R [2]. The choice of treatment is based on risk stratification and some patient-related factors (for example age, performance status, co-morbidities). Therapeutic approaches range from watchful-waiting strategy, supportive care, chemotherapy (hypomethylating agents, intensive induction chemotherapy) up to allogeneic stem cell transplantation (allo-SCT), the latter remains the only curative option [3]. According to the current recommendations, above all, allo-SCT should be offered for all fit patients with higher risk IPSS-R (high, very high and some cases from intermediate risk group—especially if score is over 3.5). Transplant strategy should also be considered for patients with lower risk IPSS-R scores, good performance and some poor risk factors (e.g. unfavorable cytogenetics, life-threatening cytopenias and high transfusion requirements). The upfront allo-SCT is recommended for patients with less than 10% marrow blasts. Patients with an increased marrow blast ≥ 10% should receive pre-transplant cytoreductive treatment (intensive chemotherapy or hypomethylating agents) [4]. Despite some advances in transplant strategies over the past decades, allo-SCT remains a high-risk procedure associated with transplant-related complications and long-term survival rate of about 30–50% [2, 5]. In this report, we present our data on the impact of patient- and disease-related factors on the outcome of allo-SCT in patients with MDS.

## Materials and Methods

The patients undergoing allogeneic stem cell transplantation in our center were retrospectively identified through the use of our institutional database of medical records. Diagnosis of MDS was made according to the 2001 World Health Organization criteria with subsequent updates [6–8] and the following categories were considered: MDS with single lineage dysplasia (MDS-SLD), MDS with multilineage dysplasia (MDS-MLD), MDS with ring sideroblasts (MDS-RS), MDS with isolated del5(q) and MDS with excess blasts 1 and 2 (MDS-EB1/2). A proportion of patients with > 10% of marrow blasts received pre-transplant cytoreductive therapy however the final decision was left to treating center. Cytogenetics was assessed at diagnosis on bone marrow cells using standard techniques. International Prognostic Scoring System (IPSS), its revised version (IPSS-R) and European Group for Blood and Marrow Transplantation (EBMT) risk score were calculated according to Greenberg et al. [9] and Gratwohl [10], respectively. The hematopoietic cell transplantation-comorbidity index (HCT-CI) was assessed according to Sorror et al. [11]. Bone marrow aspirate/biopsy was performed for response assessment at day +30, +60, +100 after transplantation and then every 6 months. Minimal residual disease (MRD) was not assessed. Molecular data were not available. The amplification of short tandem repeats (STRs) markers by polymerase chain reaction (PCR) in combination with fluorescence detection of the donor/recipient alleles by capillary electrophoresis was used for chimerism assessment [12]. Acute and chronic graft versus host disease (GvHD) were diagnosed and graded according to the standard criteria [13]. The transplantation was considered in fit patients with intermediate-2/high IPSS and/or with (very) poor risk IPSS-R or intermediate/low or unknown IPSS(R) when patient remained transfusion dependent or had other poor risk features [4]. Patients were recruited from different centers in Poland hence not all data are available. The choice of treatment (hypomethylating agent or induction-like regimen) was left to treating physician. As per our local guidelines the induction-like regimen is given to patients with blast % between 15 and 19%. Our first patient on azacitidine was recruited in 2013. All patients provided an informed consent in accordance with the Declaration of Helsinki.

### Response Criteria

Response after transplantation was estimated using International Working Group Criteria [14].

### Statistics

There were following endpoints of interest: overall and relapse-free survivals (OS, RFS), cumulative incidence for non-relapse mortality (NRM) and for relapse (CIR). Time to event was assessed from the day of transplantation. Overall survival (OS) was defined as time from day of transplant to death from any cause. Relapse-free survival (RFS) was defined as the time from stem cell infusion to disease relapse, progression or death from any cause, whichever occurred first. Non-relapse mortality (NRM) was defined as any death before clinical progression or disease recurrence; relapse is considered as competing event. Relapse incidence (RI) defined the time from transplantation to first relapse or progression; death without relapse/progression is a competing event. Patient, treatment and transplant-related data were compared by Mann–Whitney test for continuous variables and chi-square test for categorical variables. The distribution for OS and RFS were estimated using Kaplan and Meier method and compared using the log-rank test, whereas the distribution of NRM and CIR were estimated by Cumulative Incidence Function and compared using the Gray’s test. A *p* < 0.05 was considered significant. The variables tested for prognostic significance for OS, RFS, NRM and CIR included patient-related data (age, gender, MDS type, blast proportion in blood and marrow, hemoglobin concentration, platelet count, neutrophil count, serum ferritin level, IPSS-R, cytogenetics), treatment-related data (the administration of hypomethylating agents and induction regimens) and transplant-related data (donor’s age and gender, CMV status, ABO blood group, HLA compliance, the presence and grading of acute and chronic GvHD, the number of transplanted CD34 and CD3-positive cells). Proportional hazard models (Cox regression) were fitted to investigate effects of prognostic factors for OS and RFS, moreover the Fine-Gray subdistribution hazard models were used in case of NRM and CIR. Results were expressed as hazard ratio (HR) with 95% confidence interval (CI). All computations were performed with StatSoft Poland analysis software (version 12.0) and SAS version 9.4 (SAS Institute Inc., Cary, North Carolina, USA).

## Results

### Patient Characteristics

One hundred and two patients (45 females and 57 males) with MDS at median age of 47 years at diagnosis (range 18–72) underwent allo-SCT between years 2000 and 2020. Patients with different subtypes of MDS were enrolled into the study and MDS-MLD and EB2 were the most common. Data on IPSS and IPSS-R were available for 80 patients; 35 (44%) of them showed intermediate-2/high and high/very high risk category, respectively. Cytogenetics on bone marrow cells were conclusive in 80 patients; 33 (41%) individuals demonstrated diploid karyotype. Among cytogenetic abnormalities complex karyotype was most frequently observed (24%). The treatment before transplantation varied and corticosteroids and cyclosporine were most commonly used. In total, 36 patients received debulking therapy before transplantation due to increased proportion of blast cells in bone marrow (21 patients received azacitidine and 15 subjects were treated with AML-like induction: daunorubicin/cytarabine). As a result, 13 out of the 36 treated patients (36%) had < 10% blasts in BM before transplantation. The proportion of blasts was > 10% but < 19% in the remaining 23 patients. More than 50% of individuals were red blood cells (RBCs)-dependent at transplant, more than 30% of patients required regular platelet transfusion. Median blast proportion in blood and marrow at transplant was 0% (range 0–12) and 3% (range 0–19), respectively. Serum ferritin level was measured before conditioning and its median level was 1200.4 ng/ml (range 4.1–8841). Patients’ characteristics is shown in Table 1.

**Table 1:** Patient characteristics

Variable	n = 102
Gender (female/male)	45/57
Age at diagnosis, years; median (range)	47 (18–72)
MDS subtype at diagnosis, n	
MDS-SLD/RS/5q	16
MDS-MLD	47
MDS-EB1	9
MDS-EB2	30
IPSS, n^a^	
Low	11
Int-1	34
Int-2	26
High	9
IPSS-R^a^	
Very low	9
Low	17
Intermediate	19
High	21
Very high	14
Treatment for MDS, n	
Steroids	46
CsA	13
Androgens	6
ATG	3
Lenalidomide	3
TPO agonist	2
EPO	4
LD Ara-C	5
Azacitidine	21
AML-induction	15
No treatment	27
Red blood cell transfusion dependence, n	58
Platelet transfusion dependence, n	37
Time from MDS diagnosis to transplant, months; median (range)	10.9 (1.9–131)

AML, acute myeloid leukemia; Ara-C, cytarabine; ATG, anti-thymocyte globulin; CsA, cyclosporine; EB, excess of blasts; EPO, erythropoietin; IPSS, international prognostic scoring system; LD, low dose; MDS, myelodysplastic syndrome; MLD, multilineage dysplasia; SLD, single lineage dysplasia; TPO, thrombopoietin; RS, ring sideroblasts; WHO, World Health Organization

^a^Data on 80 patients

### Transplant Data

#### Baseline Characteristics of Transplanted Patients

Median recipient age was 48 years (range 18–72) whereas donors were significantly younger—32 years (range 16–67). Median time from diagnosis of MDS to transplantation was 10.9 months (range 1.9–131). Twenty seven patients were transplanted from HLA-matched sibling and 75 patients received either 10/10 HLA-matched unrelated donor (n = 60) or 9/10 HLA-mismatched grafts (n = 15). Peripheral blood was a source of stem cell for 79 patients. Reduced intensity conditioning (RIC) was used in 64 subjects whereas myeloablative regimen (MAC) was given in 38 individuals. MAC consisted of busulfan and cyclophosphamide and fludarabine-based regimens were given as RIC. GvHD prophylaxis included cyclosporine with methotrexate. Anti-thymocyte globulin (Thymoglobulin) at 5 mg/kg b.w. was given for unrelated transplantations. Low hematopoietic cell transplant-comorbidity index (HCT-CI) and high European Bone Marrow Transplant (EBMT) score were calculated in most patients.

#### Outcome of Transplanted Patients

There were two primary graft failures (PGF) and those patients proceeded to second allo-SCT from the same donor. Median time to engraftment for the remaining study population was 15 days (range 11–100). Acute and chronic GvHD developed in 61 (61%) and 19 (19%) of patients, respectively. Acute GvHD grade III–IV developed after median of 17 days (range 8–98) and was present in 20 patients. Four patients had severe chronic GvHD. Infectious complications were commonly seen early after transplantation and included 50% of transplanted population. 18 individuals developed pneumonia which resulted in septic shock and multiorgan dysfunction in 4 patients. Six patients had BKV-related hemorrhagic cystitis. CMV reactivation was demonstrated in 34 patients. Serious non-infectious complications were seen in 22 patients; 3 patients developed veno-occlusive disease (VOD), capillary leak syndrome (CLS) was seen in 2 patients and one individual had thrombotic thrombocytopenic purpura (TTP). Two patients had hemorrhagic complications; gastrointestinal bleeding and intracerebral hemorrhage. Eleven patients died within the first 30 days and 29 died during 100 days after transplantation. Fifty four deaths were noted within the first year after allo-SCT. The remaining 7 deaths occurred > 1 year after procedure. The causes of death within the first 30 days after transplantation included pneumonia (n = 8), VOD with CLS (n = 1), TTP (n = 1) and intracerebral bleeding (n = 1). Post transplant bone marrow assessment was performed in 74 patients at day +100 ± 7 days and demonstrated complete remission in 48 patients, 9 patients had stable disease, 12 patients remained transfusion dependent and 5 patients transformed into AML. All the latter patients had pre-transplant BM blast > 5%.

In total, 61 patients have died. The main causes of death included infectious complications (n = 30), steroid-resistant GvHD (n = 17), relapse with subsequent resistance to treatment (n = 9) and transformation to AML (n = 5).

**Fig. 1:**
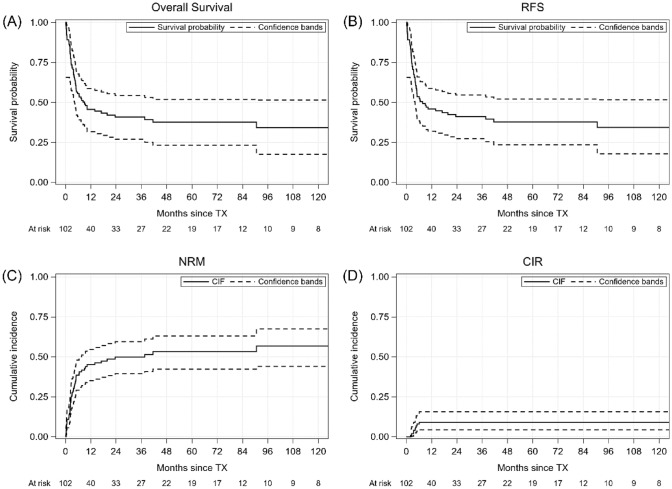
OS, RFS, NRM and CIR for allotransplanted MDS patients

41 patients are alive at last contact and present full donor chimerism. 38 patients remain in CR, 3 had CR with incomplete platelet recovery, but remained transfusion-independent. Median follow-up from diagnosis of MDS and transplantation are 27.1 months and 7 months respectively. Transplant data are summarized in Table 2. The probability of OS and relapse-free survival (RFS) were 41% at 2 years. Non-relapse mortality (NRM) and cumulative incidence of relapse (CIR) at 2 years were 49.8% and 9%, respectively (see Fig. 1). Median follow-up for survivors was 49.7 months (range 3.46–192.0).

**Table 2:** Transplant data

Variable	n = 102
Age of recipient, median; years (range)	48 (19–72)
Age of donor, median; years (range)	32 (16–67)
Year of transplant, n	
< 2010	27
2010–2015	38
> 2015	37
EBMT score; n	
Low	6
Intermediate	36
High	60
HCT-CI score; n	
Low	74
Intermediate	19
High	9
Donor type, n	
Matched related	27
10/10-HLA matched unrelated	60
9/10-HLA matched unrelated	15
Graft source	
Peripheral blood	79
Bone marrow	23
Hemoglobin level (g/dL); median (range)	9.1 (3.5–15.8)
Neutrophil count (× 10^9^/L); median (range)	1.2 (0.07–10.9)
Platelet count (× 10^9^/L); median (range)	44 (1–634)
Blast percentage in blood; median (range)	0 (0–12)
Blast percentage in bone marrow; median (range)	0 (0–19)
Ferritin level (ng/ml); median (range)^a^	1200.4 (4.1–8841)
< 1000 ng/ml; n; %	32 (44)
≥ 1000 ng/ml; n; %	41 (56)
Myeloablative conditioning, n	38
Conditioning regimen, n	
Busulfan/Cyclophosphamide	38
Treosulfan/Fludarabine	37
Busulfan/Fludarabine	18
Other	9
Number of transplanted CD34-positive cells (× 10^6^/kg); median (range)	5.05 (0.8–13.9)
Neutrophil engraftment; days, median (range)	15 (11–100)
Platelet engraftment; days, median (range)	18 (10–55)
Acute GvHD, n	
Grade I–II	41
Grade III–IV	20
Chronic GvHD, n	19
Median follow-up from transplantation, months; median (range)	7.05 (0.13–192)
Median follow-up from MDS diagnosis, months; median (range)	27.1 (5.3–204.2)

EBMT, European Blood and Bone Marrow Transplantation; GvHD, graft versus host disease; HCT-CI, hematopoietic cell transplant comorbidity index

^a^Data on 73 patients

#### Univariable (UVA) and Multivariable Analysis (MVA) of Risk Factors

##### Overall Survival

**Fig. 2:**
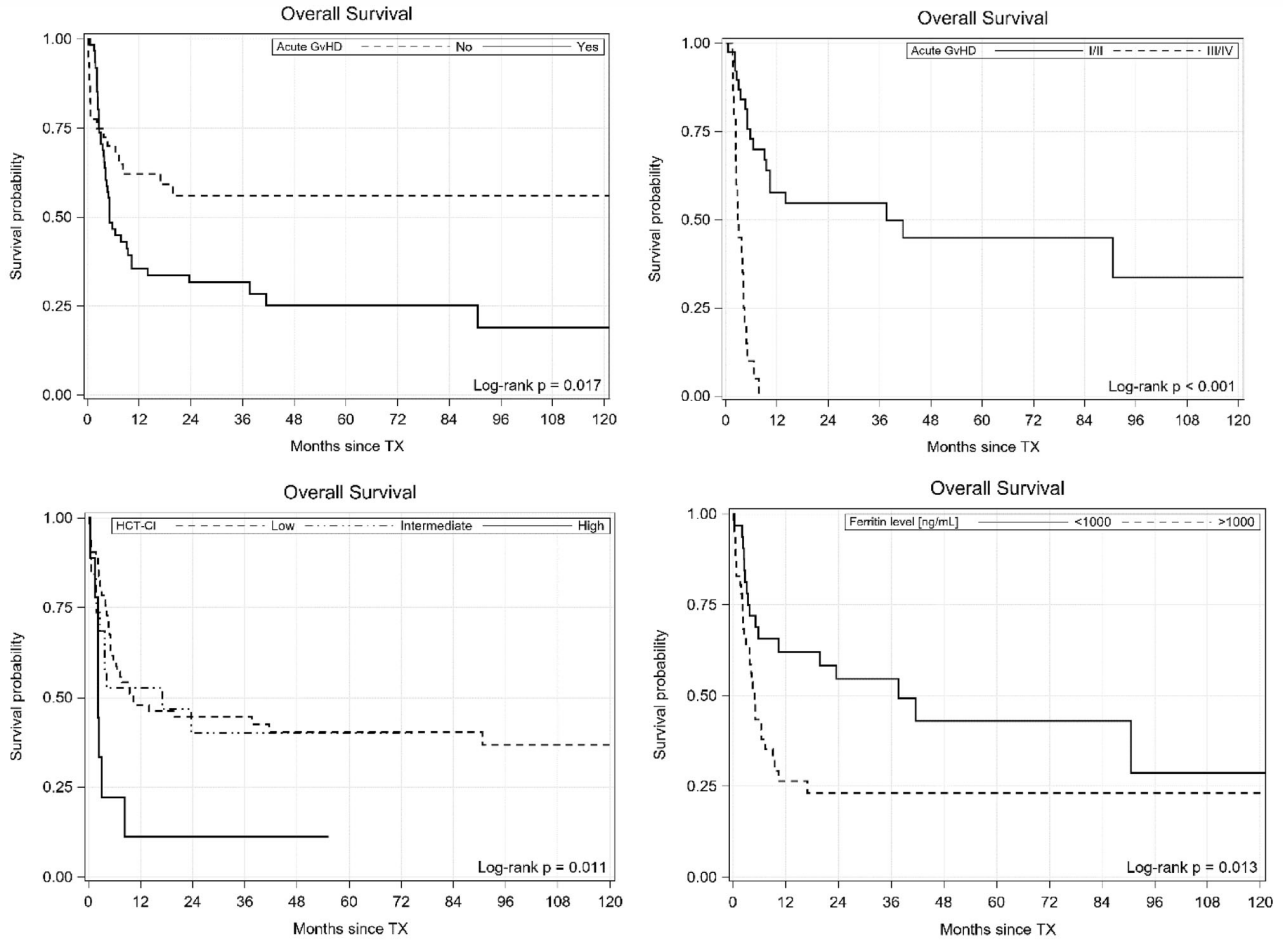
Survival outcomes according to the presence of acute GvHD, acute GvHD grading, HCT-CI and serum ferritin level

The following factors influenced OS in UVA: HCT-CI, serum ferritin level, the presence of acute GvHD, acute GvHD grading, EBMT score and year of transplantation. All but year of transplantation and EBMT score negatively influenced survival in MVA (see Fig. 2). Neither IPSS and IPSS-R nor type of conditioning, type of donor, year of transplantation and blast percentage before transplantation affected survival.

##### Relapse-Free Survival

The same set of variables as for OS influenced RFS in UVA, but statistical significance was demonstrated for HCT-CI, acute GvHD, acute GvHD grading and serum ferritin level in MVA.

##### Non-relapse Mortality

The presence of acute GvHD, acute GvHD grading and CMV reactivation had an impact on NRM in UVA, but only acute GvHD grading remained significant in MVA.

##### Cumulative Incidence of Relapse

Blast percentage in bone marrow at transplant, EBMT score and acute GvHD grading influenced CIR in UVA, but only EBMT score was significant in MVA.

Details were present in Table 3.

**Table 3:** Univariable and multivariable analysis of risk factor for OS, RFS, NRM and CIR (Cox regression)

Variable (n)	*p* value	HR (95% CI)	*p* value
*Univariable and multivariable analysis of risk factor for overall survival*			
HCT-CI	0.004	5.06 (1.06–24.1)	**0.01**
Low (74)			
Intermediate (19)			
High (9)			
Acute GvHD	0.01	8.37 (0.98–71.6)	**0.017**
Yes (61)			
No (41)			
Acute GvHD	< 0.001	8.71 (2.77–27.3)	**< 0.001**
GI-GII (41)			
GIII-GIV (20)			
Serum ferritin level	0.01	2.84 (1.09–7.27)	**0.01**
< 1000 ng/ml (32)			
≥ 1000 ng/ml (41)			
Transplant year	0.03	1.08 (0.43–2.73)	0.11
< 2010 (27)			
2010–2015 (38)			
> 2015 (37)			
EBMT score	0.06	0.98 (0.62–1.55)	0.9
Low (9)			
Intermediate (36)			
High (60)			
*Univariable and multivariable analysis of risk factor for relapse-free survival*			
HCT-CI	0.003	5.93 (1.20–29.12)	**0.008**
Low (74)			
Intermediate (19)			
High (9)			
Acute GvHD	0.02	7.59 (0.9–63.4)	**0.01**
Yes (61)			
No (41)			
Acute GvHD	< 0.001	5.97 (2.06–17.3)	**< 0.001**
GI-GII (41)			
GIII-GIV (20)			
Serum ferritin level	0.01	2.85 (1.09–7.43)	**0.01**
< 1000 ng/ml (32)			
≥ 1000 ng/ml (41)			
Transplant year	0.03	0.94 (0.36–2.47)	0.11
< 2010 (27)			
2010–2015 (38)			
> 2015 (37)			
*Univariable and multivariable analysis of risk factor for non-relapse mortality*			
Acute GvHD	0.02	19.9 (3.78–104.5)	**0.0004**
Yes (61)			
No (41)			
Acute GvHD	< 0.001	2.93 (0.54–15.7)	0.2
GI-GII (41)			
GIII-GIV (20)			
CMV reactivation	0.03	2.24 (0.85–5.9)	0.1
Yes (34)			
No (68)			
*Univariable and multivariable analysis of risk factor for cumulative incidence relapse*			
Blast % in bone marrow at transplant	0.03	1.02 (0.89–1.17)	0.7
EBMT score	< 0.001	0.63 (0.02–16.51)	**< 0.001**
Low (9)			
Intermediate (36)			
High (60)			
Acute GvHD	< 0.001	16.78 (1.69–166.28)	0.19
GI–GII (41)			
GIII–GIV (20)			

Bold indicates statistical significance

## Discussion

Allo-SCT remains the only curative therapeutic approach for patients with MDS, however the procedure is related to significant morbidity and mortality. A weighed assessment of benefit and risk of transplantation should be done before taking a final decision. Lower risk patients can experience long-term survival with stable disease and they would not benefit from an early SCT. These patients have better life expectancy when transplantation is delayed until disease progression or treatment failure [15–17]. Nonetheless, allo-SCT should be considered in patients with good performance and poor prognostic factors defined as poor-risk cytogenetic characteristics, high transfusion burden or life-threatening cytopenias [18]. Patients with higher risk MDS have a poor prognosis and increased risk of transformation to acute leukemia with the median OS < 2 years. However, not all these patients can be candidates for allogeneic transplant due to advanced age or co-morbidities.

In our study, the estimated 2-year OS was 41% and our findings were similar to those reported by others [19–21]. In a large study of the European Group for Blood and Marrow Transplantation (EBMT), the 3-year disease-free survival of 712 patients transplanted for primary MDS from 1983 to 1998 was 37% [22]. Other large registry reported overall survival rates of 42% at 3 years on 452 cases of HLA-identical sibling donor transplantation [23]. The probability of survival of 510 patients with MDS who underwent unrelated donor bone marrow transplantation at 2 years was 30% [24]. The introduction of RIC regimens provided the possibility of transplantation for older patients with 2–4-year OS rates ranging from 23 to 75% [25–29]. Better results were reported in a more recent prospective study—overall survival at 2 years was 76.3% after RIC and 63.2% after myeloablative conditioning [30]. Although allo-SCT is potentially curative, it carries a high risk of non-relapse mortality (NRM). The incidence of NRM ranges from 36% even to 66% [22–30]. In our study NRM was in line with those provided by others and reached ~ 50% at 2 years.

We analyzed impact of pretransplant and transplant factors affecting patients’ survival. The EBMT risk score provides a simple tool to assess chances and risks of allo-SCT for an individual patient [10]. In our study EBMT score tended to affect survival in univariable analysis but it was found not to influence survival in multivariable analysis—OS at 2 years for patients with low EBMT score was 83% when compared with 34% and 29% for intermediate and high-risk EBMT scores, respectively. Our findings are similar to Lozano study, in which 9741 patients with MDS were included [31]. In that report the EBMT score accurately predicted OS at 5 years—50%, 41% and 31% for high, intermediate and low risk scores respectively. It also correlated with incidence of treatment-related mortality. No differences in the relapse risk among EBMT score groups were observed.

Our results demonstrated that iron overload measured by pre-transplantation serum ferritin level had a negative impact on OS after allo-SCT. These findings are in agreement with the previous studies [32–34]. Moreover, some of these reports have also showed that serum ferritin level was found to be a risk factor for development of severe infections and acute GvHD. Cremers et al. demonstrated that patients with serum ferritin level ≥ 1000 ng/ml had a 14% lower 2-year survival, higher NRM and relapse incidence than patients with serum ferritin level below 1000 ng/ml [35]. Pretransplant serum ferritin level has also been included in a prognostic score for patients with acute leukemia or MDS undergoing allo-SCT [36]. Armand et al. proposed a scoring system based on 5 variables (age, disease, stage at transplantation, cytogenetics, and pre-transplantation ferritin), which divided patients into 3 groups with 5-year overall survival of 56% (low risk), 22% (intermediate risk), and 5% (high risk). However, serum ferritin level is also widely recognized as an inflammatory marker and its role as a marker of iron overload should be considered with some caution.

Another factor that influenced OS was the occurrence of acute GvHD—a 2-year OS was significantly worse in patients who developed acute GvHD when compared with those who did not (31% vs 56%). GvHD was also one of the most common cause of death in transplanted patients (17 out of 61 died patients). Moreover, patients presented with grades I–II acute GvHD fared much better than those with grades III–IV (56% vs 0% at 2 years; HR 8.71; 95% CI 2.77–27.3; *p* < 0.001). Grade III–IV acute GvHD developed in 20% patients (grades II–IV in 37%) and our results were in line with those presented by other authors [23, 30, 31, 37]. In Oran study, the cumulative incidence of grade II–IV acute GvHD was 39% and development of grade II–IV acute GvHD was associated with significantly shorter survival [25].

Surprisingly, patients transplanted before 2010 fared much better in UVA than those who received graft between 2010 and 2015 (52% vs 29% at 2 years, respectively; data not published), however this finding was not confirmed in MVA.

In our study, transformation to AML or relapse occurred in 5 (4.9%) and 9 (8.8%) patients, respectively. All patients who progressed to leukemia had pre-transplant BM blast > 5% and 90% received cytoreduction therapy without achieving CR after treatment. It was demonstrated that treatment resistance before transplantation was a poor prognostic factor [16, 38–41]. The cumulative incidence of relapse at 2–4 years differs between reports—ranging from 6% to even to 46% in the high risk groups [23, 25, 41–43].

Our report has several limitations that should be acknowledged. This study was retrospective and included data from a single institution registry. Nonetheless, our report has demonstrated that allo-SCT is a potentially curative treatment option for proportion of patients with MDS. On the other hand, one should be aware of severe post-transplant complications including infections and steroid-resistant GVHD. A large prospective studies should be initiated to better define factors influencing survival after allo-SCT for MDS.
